# Cell type-dependent modulation of senescence features using Weo electrolyzed water

**DOI:** 10.18632/aging.205789

**Published:** 2024-04-30

**Authors:** Brenda L. Court-Vazquez, Shirley A. Arroyo-Vizcarrondo, Jonathan A. Poli, Lara Nyman, Kelly Halderman, Anthony Ginter, Pierre-Yves Desprez

**Affiliations:** 1 Weo LLC, Research and Development Department, Miami, FL 33136, USA; 2 California Pacific Medical Center, Research Institute, San Francisco, CA 94107, USA

**Keywords:** cellular senescence, senescence-associated secretory phenotype, oxidative stress, lung fibroblasts, breast cancer cells, senomorphic

## Abstract

Electrolyzed-reduced water has powerful antioxidant properties with constituents that scavenge reactive oxygen species (ROS), which are known to be produced by several intrinsic and extrinsic processes. When there is an imbalance between ROS production and antioxidant defenses, oxidative stress occurs. Persistent oxidative stress leads to cellular senescence, an important hallmark of aging, and is involved in several age-related conditions and illnesses. This study aims to investigate whether Weo electrolyzed water (WEW) could modulate the phenotype of senescent cells. We compared normal human lung fibroblasts (BJ) and breast cancer cells (T47D) treated with hydrogen peroxide (H_2_O_2_) to induce senescence. We assessed the molecular impact of WEW on markers of cellular senescence, senescence-associated secretory phenotype (SASP) factors, and stress response genes. Treatment with WEW modulated markers of cellular senescence, such as the senescence-associated β-galactosidase (SA-β-gal) activity, EdU incorporation and p21 expression, similarly in both cell types. However, WEW modulated the expression of SASP factors and stress response genes in a cell type-dependent and opposite fashion, significantly decreasing them in BJ cells, while stimulating their expression in T47D cells. Reduction in the expression of SASP factors and stress-related genes in BJ cells suggests that WEW acts as a protective factor, thereby reducing oxidative stress in normal cells, while making cancer cells more sensitive to the effects of cellular stress, thus increasing their elimination and consequently reducing their deleterious effects. These findings suggest that, due to its differential effects as a senomorphic factor, WEW could have a positive impact on longevity and age-related diseases.

## INTRODUCTION

Cellular senescence is a multifaceted phenomenon characterized by the cessation of cell division. It was first reported by Hayflick and Moorhead decades ago when they observed how normal cells proliferate in culture, but only for a finite period [1]. Other characteristics that appear in senescent cells include an enlarged and flattened phenotype, increased expression of the senescence-associated β-galactosidase (SA-β-gal) [2], and increased expression of a secretome, i.e., the senescence-associated secretory phenotype (SASP) [3, 4].

Senescence plays an important physiological role during normal development or upon acute damage, contributing to tissue remodeling [5] and wound healing [6]. Moreover, senescent cells contribute to tumor suppression by ensuring that potentially dysfunctional, damaged, or untransformed cells do not carry on their genomes to the next generation [7]. The secretion of specific factors and cytokines via the SASP informs the immune system to initiate the clearance of senescent cells and stimulates the damaged tissue to heal.

However, cellular senescence can also result in detrimental effects to the tissue or to the entire organism. In older individuals, the process of clearance is dysregulated, and senescent cells accumulate, contributing to chronic inflammation and several age-related disorders including cancer [8]. Given these antagonistic effects, cellular senescence is regarded as a double-edged sword with both beneficial and detrimental effects on health and disease, and thereby considered to be an example of evolutionary antagonistic pleiotropy [9, 10]. Studies have demonstrated that mitotic cells can become senescent in response to several internal and external stressors, including telomere shortening, mitochondrial deterioration, chromatin disruption, DNA damage response (DDR), oncogene activation, or oxidative stress [11, 12].

Oxidative stress is defined as an imbalance in the production of reactive oxygen species (ROS) relative to antioxidant defenses [13]. While ROS can be produced by both intrinsic and extrinsic factors and are known to play essential roles in several biological processes, excessive production can cause damage to cellular macromolecules, such as DNA, proteins, and lipids, which are considered major factors in the development of diseases [13].

The negative effects caused by ROS can be counterbalanced by antioxidant defenses [13]. Several investigations have reported positive effects of electrolyzed reduced water (ERW) on human health [14]. ERW can be produced by electrolysis, a process by which electric current is passed through a substance to effect a chemical change, and this change causes the substance to lose or gain an electron (oxidation or reduction). This process also splits water into its two main components: oxygen and hydrogen gas. Due to its antioxidant properties, electrolyzed water has been shown to reduce the damage caused in cells by oxidative stress and inflammation [15]. Moreover, Tanaka et al. investigated the effects of alkaline electrolyzed water (AEW) for individuals with gastrointestinal symptoms. The study showed that a 4-week period of consuming this type of water resulted in a normalization in their intestinal system [16].

While literature to date has identified molecular hydrogen as the key bioactive in electrolyzed water, the electrolysis system, conditions, and materials used can have a large impact on the product bioactivity including the types and concentrations of bioactives created. A new patented technology developed by Weo which uses boron-doped diamonds to electrolyze water could prove beneficial to mitigate the effects of cellular senescence caused by oxidative stress on different tissues. Using this novel technology to produce a unique water made up of blended properties and components, including mixtures and combinations of dissolved molecular oxygen (O_2_), molecular hydrogen (H_2_), antioxidants, and ROS may provide new avenues for senescence-related interventions. In addition to the production of both oxygen and hydrogen species, the water appears to undergo structural and physical changes that could be associated with health benefits.

The focus of this study was to utilize two different cell types, human normal fibroblasts and human breast cancer cells, to investigate the impact of Weo electrolyzed water (WEW) on markers of cellular senescence, inflammation, and stress response genes.

## RESULTS

### Weo electrolyzed water (WEW) reduces cellular senescence in normal fibroblasts and cancer cells

To investigate the effects of WEW at different charges and time courses on normal human BJ fibroblasts and human T47D breast cancer cells that were subjected to oxidative stress, we first assessed the expression of some senescence-associated biomarkers. As expected, exposure to H_2_O_2_ in both non WEW-treated proliferating cell types led to a change in cell morphology, enhanced senescence-associated-β-galactosidase staining (SA-β-gal), decreased rate of DNA synthesis, inhibition of cell proliferation, and over-expression of the cell cycle inhibitor p21, when compared to cells that were not induced to senesce.

**Figure 1:**
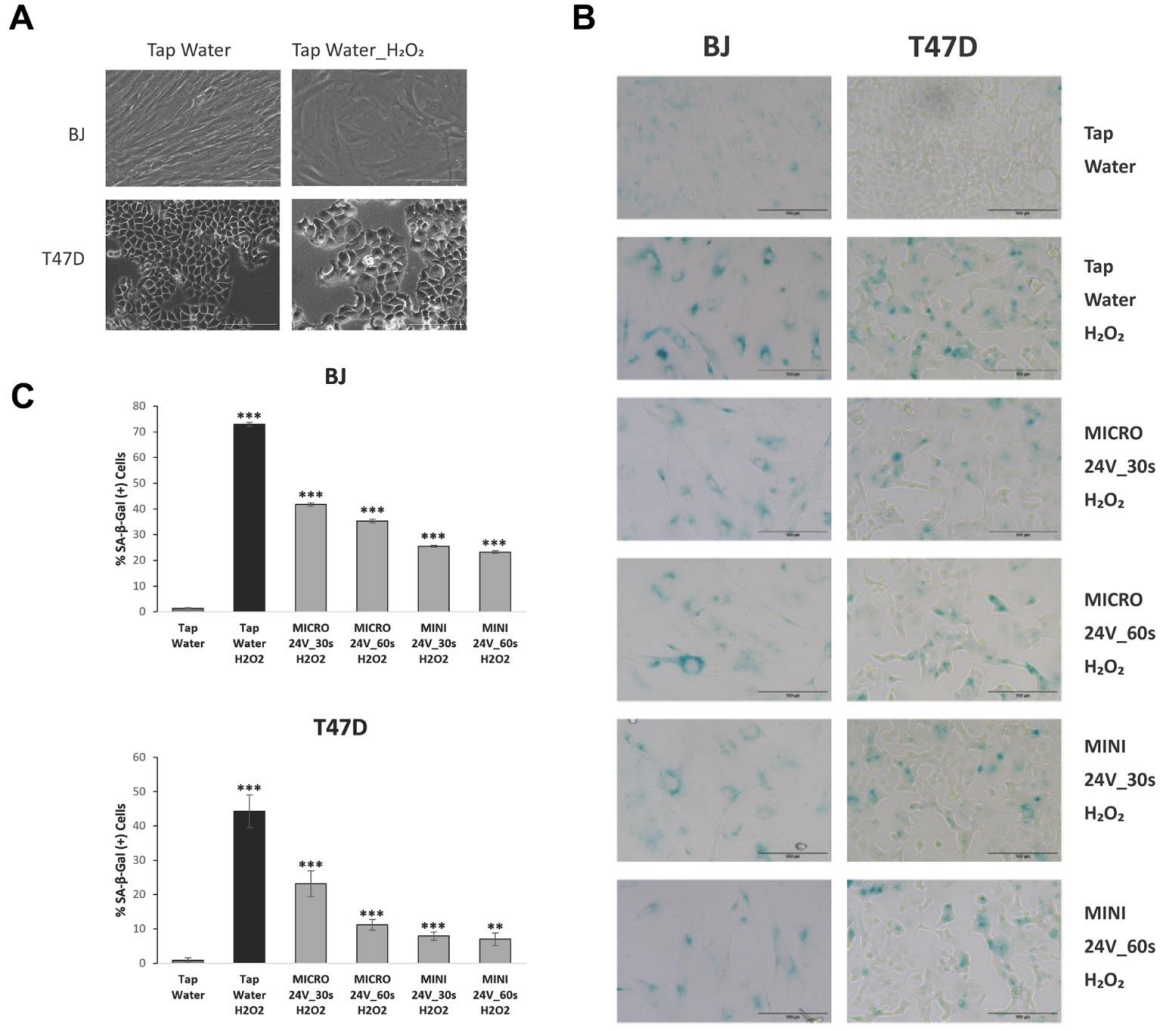
**WEW decreases cellular senescence in human fibroblasts and cancer cells.** Cells were treated with WEW and induced to senesce with H_2_O_2_. (**A**) Images of morphological changes during oxidative stress induction of senescence in BJ and T47D cells. Both cell types adopted a flattened and enlarged morphology. (**B**) SA-β-gal staining of senescent fibroblasts and cancer cells treated and non-treated with WEW. Images were taken using an inverted phase contrast microscope after SA-β-gal activity was detected by incubating the fixed cells in SA- β -gal solution at 37° C. (**C**) SA- β -gal activity was assayed by manually scoring SA-β-gal positive cells. Results are expressed as mean values (+SEM) of cell count in one of three independent experiments. Significance was calculated by a two-tailed Student’s *t* test. *P < 0.05, **P < 0.01, ***P < 0.001

**Figure 2:**
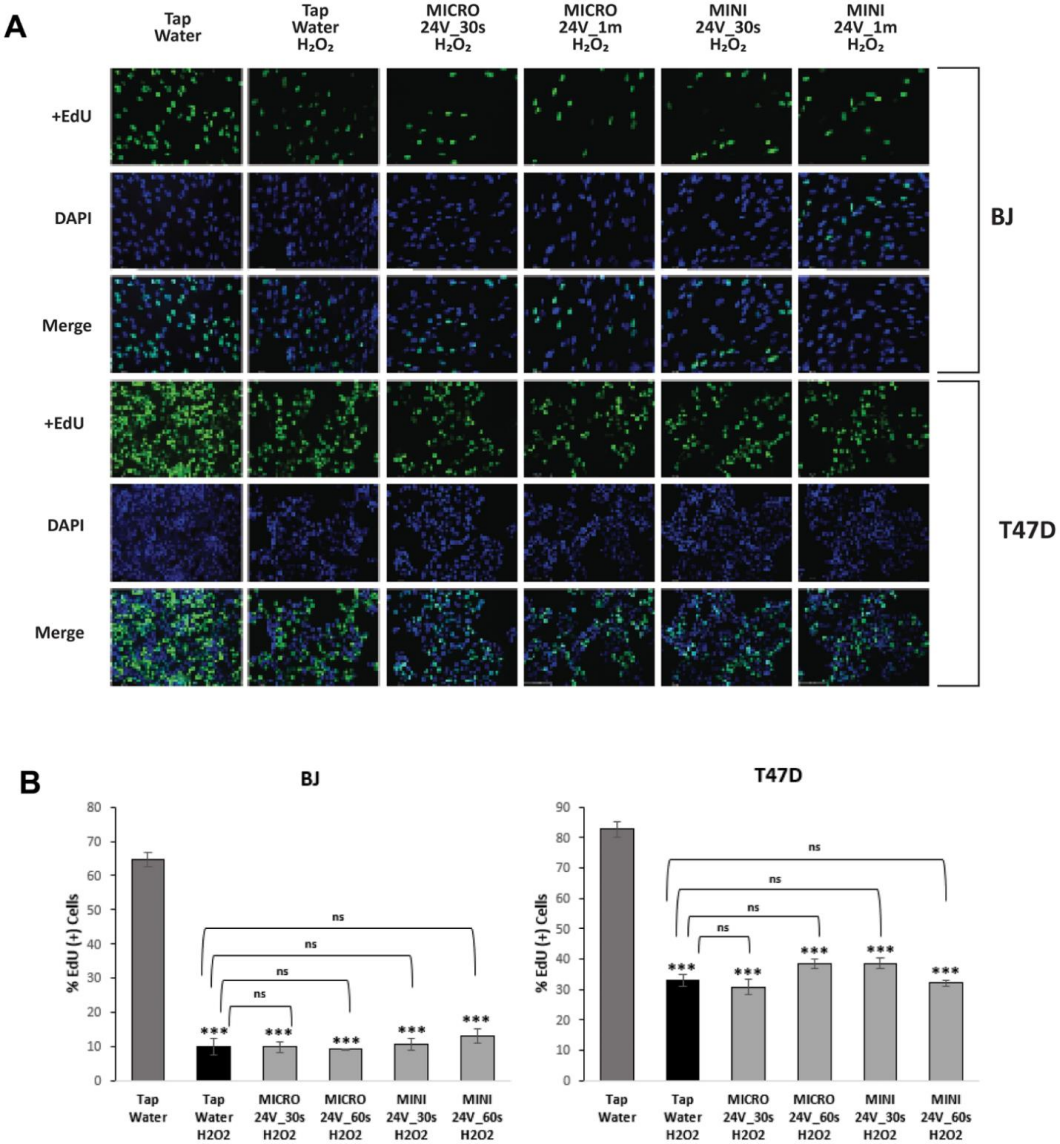
**Inhibition of DNA synthesis in H_2_O_2_-induced BJ and T47D.** Cells were treated with H_2_O_2_ to induce senescence. (**A**) Detection of EdU incorporated into the DNA of senescent and non-senescent fibroblasts and cancer cells treated or not with WEW. DNA synthesis was evaluated by immunofluorescence staining using EdU, and its incorporation was detected with Alexa Fluor 488 (green). Counterstaining with DAPI displays the localization of the nuclei (blue). (**B**) The results are expressed as a percentage of EdU incorporation after oxidative stress senescence induction and treatment with WEW or tap water. Results are given as mean ±SEM of five different fields, and significance was calculated by a two-tailed Student’s *t* test. *P < 0.05, **P < 0.01, ***P < 0.001; ns: non-significance.

Figure 1A shows representative phase contrast microscope images of BJ and T47D cells confirming an enlarged and flattened cell morphology characteristic of cellular senescence. H_2_O_2_-induced cells in non-electrolyzed water were used as controls for both cell types in this study. When comparing WEW-treated and tap water-treated H_2_O_2_-induced senescent BJ and T47D cells, microscopic analysis of SA–β–gal staining revealed a decreased SA-β-gal activity in both cell types (Figure 1B, 1C). Moreover, immunofluorescence analysis of the incorporation of 5-ethynyl-2’-deoxyuridine (EdU) showed that a low percentage of BJ and T47D cells incorporated EdU after senescence induction (Figure 2A, 2B).

**Figure 3:**
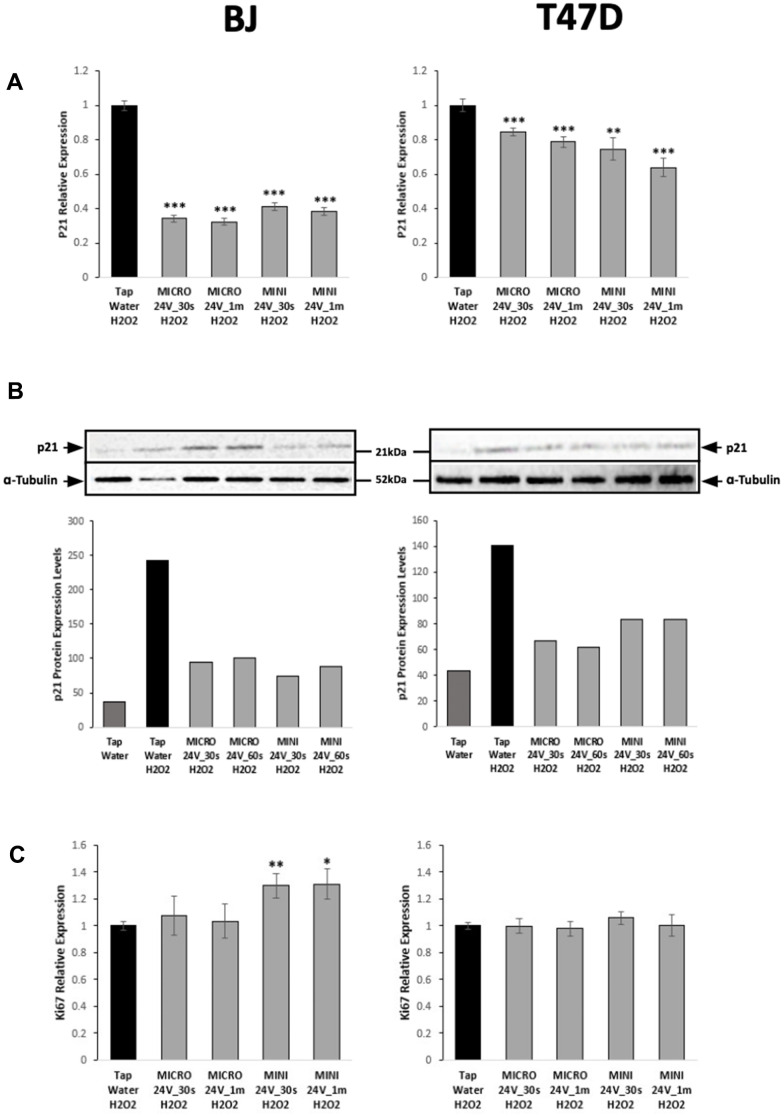
**WEW significantly reduces the expression of p21 but does not affect proliferation of BJ and T47D cells.** (**A**) Relative mRNA level of the p21 gene in BJ senescent cells and T47D senescent cells treated with WEW compared to senescent cells treated with tap water. (**B**) Protein level expression of p21 determined by western blot analysis (top panel: picture of the blots; bottom panel: quantification). Tubulin was used as internal loading control. (**C**) Relative mRNA level of the Ki67 gene in BJ senescent cells and T47D senescent cells treated with WEW compared to senescent cells treated with tap water. The graphs represent the mean ±SEM from three independent experiments. Significance was calculated by a two-tailed Student’s *t* test. *P < 0.05, **P < 0.01, ***P < 0.001.

Elevated p21, one of the most well-established senescence markers, and reduced Ki67, a cell proliferation marker, are associated with the induction of senescence. To analyze the expression of both markers, cDNAs were generated from RNA extracted from H_2_O_2_-treated BJ and T47D cells, further treated or not with WEW, and RT-PCR was performed. Our results revealed that WEW significantly downregulated p21 gene expression in both cell types (Figure 3A), particularly in normal fibroblasts, which was confirmed at the protein level (Figure 3B). However, even though WEW increased the expression of Ki67 in normal fibroblasts, WEW didn’t have any significant effect on its expression in cancer cells as shown in Figure 3C.

### WEW modulates the expression of a SASP factor in a cell type-dependent fashion

**Figure 4:**
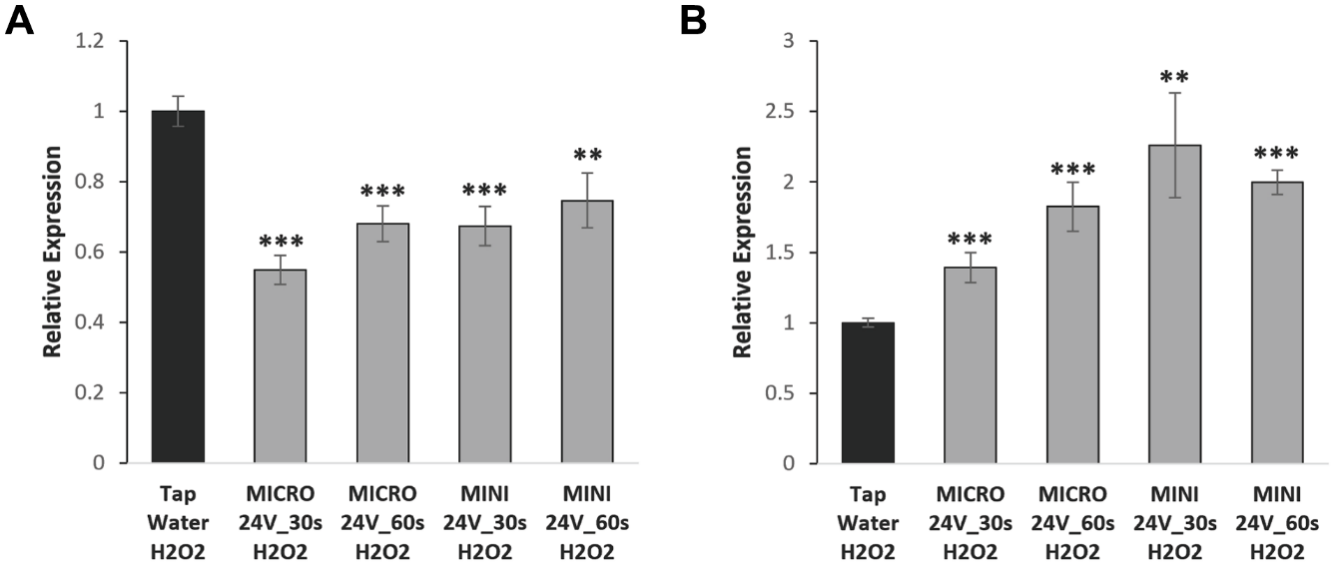
**WEW modulates the expression of CXCL-1 in a cell type-dependent fashion.** CXCL-1 gene expression was analyzed using qPCR. Relative mRNA level of CXCL-1 gene in BJ senescent cells (**A**) and T47D senescent cells (**B**) treated with WEW and compared to senescent cells treated with tap water. The graphs represent the mean ±SEM from three independent experiments. Significance was calculated by a two-tailed Student’s *t* test. **P < 0.01, ***P < 0.001

A common characteristic of cellular senescence is the upregulation of the senescence-associated secretory phenotype (SASP), which includes interleukins, chemokines, and extracellular matrix components [17]. Depending on the cell type investigated, some SASP factors will be more highly expressed than others. We examined the expression of CXCL-1, a chemokine that plays an important role in regulation of immune and inflammatory responses [18]. We assessed if WEW could modulate the expression of this SASP factor after oxidative stress induction, and we observed a substantial difference in the cell-specific regulation of this gene (Figure 4). The expression of CXCL-1 in senescence-induced BJ cells treated with WEW was significantly downregulated compared with tap water-treated control cells. However, for the T47D cancer cells, even though WEW did not increase senescence per se, a significant upregulation of the SASP factor CXCL-1 was observed.

### WEW modulates the expression of stress response genes in a cell type-dependent manner

**Figure 5:**
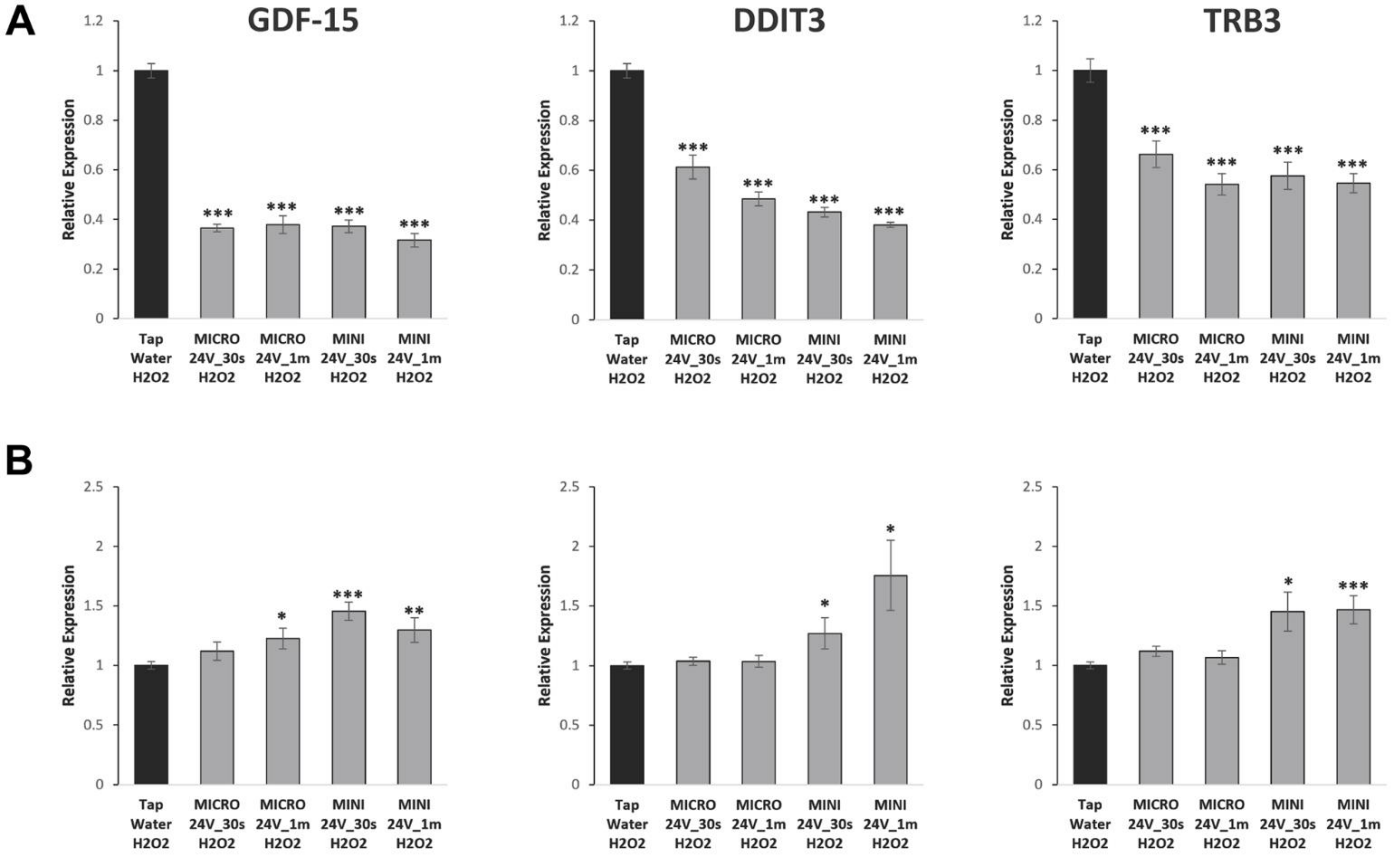
**Stress response genes are modulated by WEW in a cell type-dependent manner.** Stress response genes expression was analyzed using qPCR. Relative mRNA level of GDF-15, DDIT3, and TRB3 in BJ senescent cells (**A**) and T47D senescent cells (**B**) treated with WEW and compared to senescent cells treated with tap water. The graphs represent the mean ±SEM from three independent experiments. Significance was calculated by a two-tailed Student’s *t* test. *P < 0.05, **P < 0.01, ***P < 0.001.

We previously obtained some RNA-Seq results to screen for genes potentially regulated by WEW, and we determined that several stress response genes, such as GDF-15, DDIT3 and TRIB3, were significantly modulated (data not shown). GDF-15 has recently been described as an aging marker, and the expression of DDIT3 and TRIB3 genes is similarly modulated during stress response [19–23]. The expression of these stress response genes in BJ fibroblasts was downregulated in cells induced to senescence/stress with H_2_O_2_ and then consequently treated with WEW, compared with tap water-treated cells (Figure 5). On the other hand, an upregulation of the same genes was observed in stress-induced breast cancer T47D cells treated with WEW. These results suggest that WEW triggers gene modulation in stressed cells, which is cell strain-specific.

## DISCUSSION

The focus of this study was to determine the effects of WEW treatment in cells exposed to oxidative stress triggering cellular senescence. H_2_O_2_, our oxidative stress model, is the most used inducer of senescence in culture and an endogenous source of cellular oxidative stress [24]. Here, we first confirmed the induction of cellular senescence in human BJ and T47D cells by assessing a variety of senescence-associated biomarkers: change in cell morphology, activity of the lysosomal enzyme SA-β-gal, level of DNA synthesis, loss of proliferative potential, levels of expression of the cell cycle inhibitor p21, and expression of a SASP factor and stress response genes.

After a week of H_2_O_2_ exposure, BJ and T47D cells developed morphological changes characteristic of cellular senescence. However, WEW-treated senescent-induced BJ and T47D cells displayed a reduction in SA-β-gal activity when compared to controls. Accordingly, our study also shows that the marker of senescence, p21, was significantly downregulated in both cell types (normal and transformed). These findings suggest that WEW has a positive impact on the reduction of markers of senescence, one of the main hallmarks of aging and a major cause of age-related diseases, by reversing the senescent phenotype in both cell types investigated.

On the other hand, Ki67 was modulated differently in both cell types. Normal BJ cells treated with a higher charge of WEW showed a slight upregulation of the Ki67 gene, confirming the anti-senescent effects using WEW. Therefore, WEW may slightly stimulate the proliferative activity of normal human cells on top of reducing their senescent phenotype. However, we didn’t detect any significant difference in Ki67 expression in senescent T47D cells treated with WEW versus cells treated with tap water. Therefore, even though cellular senescence was reduced in breast cancer cells, the absence of WEW effects on their proliferative activity could be beneficial to prevent further tumor aggressiveness.

Fluorescence analysis of the incorporation of 5-ethynyl-2’-deoxyuridine (EdU) showed that a low percentage of normal BJ and tumorigenic T47D cells incorporated EdU after senescence induction, which is consistent with previous reports demonstrating that both cell types undergo growth arrest when induced to senesce [25]. Even though the two cell types did not display any difference in % EdU between Tap Water H_2_O_2_ versus Weo Electrolyzed Water H_2_O_2_, as described above, Ki67 expression was slightly elevated in WEW-treated normal cells compared to tumorigenic cells. However, this small difference was only detected using the MINI unit and not the MICRO unit. The MINI unit modifying the water more extensively than the MICRO unit, the potentially beneficial effects on the behavior of normal versus tumor cells (increasing ability of normal cells to divide) could be of interest for our overall approach.

Cellular senescence is known to influence the tissue microenvironment due to the secretion of SASP factors, which develop when senescent cells have undergone changes in protein expression and secretion. A large-scale characterization of the SASP was previously described by Coppé et al. (2008). Many of these factors, such as CXCL-1, play an important role in inflammation serving as chemoattractant for immune cells to fight infection. Here we report that WEW treatment in senescent-induced normal cells reduced the expression of CXCL-1. Conversely, senescent cancer cells treated with WEW exhibited an opposite effect on the expression of the pro-inflammatory gene. This finding agrees with other studies demonstrating that the SASP is specific and varies depending on cell type [17, 25–26]. However, we show here the same treatment can have opposite effects whether cells treated are normal or cancerous.

Senescence is a type of stress response, and, when cells enter a stress period, certain genes are activated to minimize the negative effects of the stress [27]. These stress response genes help the cells to adapt to their microenvironment to avoid cell death. Previously to this investigation, WEW-treated and control samples were sent for global gene readout using the RNA-Seq technique, and we determined that GDF-15, DDIT3 and TRIB3 were significantly modulated by WEW. GDF-15, a well-characterized gene involved in various processes, encodes a protein that acts as a pleiotropic cytokine involved in stress response upon injury, inflammation, or tissue hypoxia [21–23]. DDIT3 and TRIB3 genes are activated similarly upon stress, which in some circumstances leads to apoptosis. In normal senescent BJ fibroblasts treated with WEW, we detected a downregulation of the three stress response genes. Conversely, an upregulation of the same genes was observed in T47D cells, which could have an opposite effect in transformed versus normal cells. As for the SASP factor CXCL-1, WEW again causes gene modulation in a cell-dependent fashion.

Overall, we suggest that, depending upon the tissue of interest and cell function, WEW may be able to modulate important genes for beneficial endeavors, which could be highly relevant to health and metabolism in animals and humans. In normal human cells, a reduction in the expression of pro-inflammatory SASP factors and stress-related genes by WEW could correlate with an attenuation of oxidative stress, therefore helping in the conservation of tissue integrity. Conversely, an upregulation by WEW of inflammatory factors and stress-related genes in cancer cells could trigger the activation of immune response and/or apoptosis, which could be important for the body’s ability to eliminate deleterious cells.

In conclusion, we have shown here that the new technology developed by Weo, WEW, could attenuate the overall process of cellular senescence in both normal BJ fibroblasts and cancer T47D cells. However, and importantly, WEW modulates the expression of the SASP factor CXCL-1 and some stress response genes in a cell type-dependent fashion. While our investigation was based on oxidative stress induction to trigger cellular senescence, it remains to be determined if WEW has the same effects on cells induced to senescence by other means. Further studies need to be conducted to understand the precise molecular mechanism effects of WEW in different cell types and age-related conditions.

## MATERIALS AND METHODS

### Weo electrolyzed water preparation

Weo Electrolyzed Water (WEW) was produced using boron doped diamond-based water electrolysis technology (Weo LLC, Miami, FL, USA). The technology consists of two silicone electrodes with 2-3 microns of diamond layered on a single side. The electrodes are enclosed in an electrolytic cell where the diamond sides of the electrodes face one another, separated by 1 mm of distance. The other 5 sides of the electrode are sealed in thermoplastic housing and silicone rubber gaskets. The opposite side of the diamond side is electrically connected to a DC power source supplying 0-48VDC. Supplying a DC voltage induces a positive or negative polarity in the respective electrode, and the polarity is alternated every 30 seconds.

Unlike typical Hydrogen Water Bottles (HWB) in the marketplace, Weo’s technology does not separate the anodic and cathodic compartments with a Proton Exchange Membrane (PEM). Instead, both oxidative and reductive chemical species are present in the water during electrolysis. Two different sized electrode pairs were tested, Unit 1 having an active electrode area of 5.76 cm^2^ x 2 (micro unit) and Unit 2 having an area of 12.5 cm^2^ x 2 (mini unit). With all other conditions held constant (water conductivity, voltage, volume, duration), a larger electrode area can generate more chemical reactions, a higher charge, and different concentrations of oxygen and hydrogen species generating a different palette of bioactive species. A total of 500 ml of 37° C filtered tap water solution was prepared just before use for all experiments, and water was circulated through the electrolytic cell at a flow rate of 0.5LPM. During the experiments the current was kept between 4-8A by adjusting the voltage.

To prepare the WEW based media, DMEM or RPMI-1640 powdered media (GIBCO) and sodium bicarbonate (NaHCO_3_; MediaTech) were weighed according to manufacturer’s instructions and added to 100 ml of electrolyzed water. WEW sample’s pH was adjusted to 7.0-7.4 by adding 1N HCl (Fisher) and filtered through a 0.2 μm membrane filter. Electrolyzed water samples were used immediately after preparation.

### Cells

Normal human BJ fibroblasts and human T47D breast cancer cells were obtained from ATCC. Fibroblasts were cultured in Dulbecco’s Modified Eagle Medium (GIBCO) with 10% heat inactivated fetal bovine serum (FBS) (GIBCO) in the absence of any added antibiotic. Cancer cells were cultured in RPMI-1640 medium (GIBCO) with 10% FBS in the absence of any added antibiotic as well. Both cell types were maintained in T75 flasks (CytoOne) at 37° C in a humidified incubator with 5% CO_2_. To induce senescence by oxidative stress, cells were harvested when ~80% confluent using 0.25% Trypsin-EDTA (GIBCO). Following resuspension in their respective media, 1 ml of the cell suspension was counted and measured using a Scepter 3.0 cell counter (Millipore SIGMA).

BJ cells were seeded at 1x10^5^ cells per well on 6-well plates or at 1x10^4^ cells per well on 24-well plates, and T47D cells were seeded at 0.5x10^5^ cells per well on 6-well plates or at 0.5x10^4^ cells per well on 24-well plates. After 24h incubation, DMEM was replaced with WEW (or tap water) and fibroblasts were induced to senescence with 25 μM H_2_O_2_ for 2h, medium was replaced with WEW (or tap water) without hydrogen peroxide and cells incubated for 48h; an induction for 2h was repeated and assays were performed after 48h. After replacing RPMI-1640 and treatment with freshly made WEW media (or tap water) for 3d, cancer cells were induced to senescence with 100 μM H_2_O_2_ and assays were performed after three days of induction.

### SA-β-Gal staining

Fibroblasts and cancer cells were seeded on 6-well plates, treated with WEW (or tap water) and induced to senescence with H_2_O_2_ as described above. SA-β-gal activity was determined using the senescence β-galactosidase staining kit (Cell Signaling Technology) according to manufacturer’s instructions. Cells were incubated at 37° C without CO_2_ and monitored using a phase contrast light microscope (Leica) until the staining became visible. A minimum of 500 cells were counted to determine the percentage of SA-β-gal-positive cells.

### EdU incorporation

Cells were treated with WEW (or tap water) and induced to senescence on 24-well plates containing coverslips as described above. Cells were then washed twice with PBS (GIBCO), fixed with 3.7% formaldehyde (VWR), and permeabilized with 0.5% Triton X-100 (Millipore). To assess the rate of DNA synthesis, the Click-iT chemistry was performed by reacting EdU with Alexa Fluor 488 (ThermoFisher Scientific) for 30 min at dark. Coverslips were washed twice in PBS prior to mounting them with ProLong™ Gold Antifade Mountant with DAPI (Molecular Probes). Images were obtained using a phase contrast fluorescence microscope (Leica).

### Quantitative real time polymerase chain reaction (RT-PCR)

Both cell types were seeded, treated with WEW or tap water, and induced to senescence in triplicates onto 6-well plates prior to trypsinization with 0.25% Trypsin-EDTA (GIBCO) and centrifugation at 300 *g* for 5 min. Cell pellets were suspended in cell lysis buffer (RLT Plus buffer; QIAGEN) containing 10:1000 β-mercaptoethanol and homogenized by vortexing. Total RNA was isolated using the RNeasy Plus Mini kit (QIAGEN) according to the manufacturer’s protocol. RNA concentrations from each sample were quantified using the NanoDrop One^C^ spectrophotometer, and samples were normalized to ~1 μg prior to reverse transcription. cDNA was synthesized using the qScript Ultra SuperMix (Quantabio). Quantitative reverse transcription reactions were done in triplicate using FastSYBR Mixture - Low ROX (CWBIO) and analyzed using the QuantStudio 3 sequence detector. Primer sets (Integrated DNA Technologies) for human genes used are listed in Table 1. Fold changes were calculated by the delta-delta Ct method (fold = 2ddCt). Samples were normalized to the cycle threshold value obtained during exponential amplification of actin.

**Table 1:** RT-qPCR primers.

**Human primers**	**Forward (5’→3’)**	**Reverse (5’→3’)**
*CDKN1A (p21)*	TCACTGTCTTGTACCCTTGTGC	GGCGTTTGGAGTGGTAGAAA
*Ki67*	GAAAGAGTGGCAACCTGCCTTC	GCACCAAGTTTTACTACATCTGCC
*CXCL-1*	GCTGAACAGTGACAAATCCAAC	CTTCAGGAACAGCCACCAGT
*GDF-15*	GAAGATTCGAACACCGACCTC	CCCGAGAGATACGCAGGT
*DDIT3*	AGCGACAGAGCCAAAATCAG	CAATGACTCAGCTGCCATCT
*TRB3*	GGCACTGAGTATACCTGCAAG	GAGTGAAAAAGGCGTAGAGGA

Forward and reverse primers for RT-qPCR using FastSYBR mixture technology as indicated.

### Western blotting

Cells were seeded, treated with WEW or tap water, and induced to senescence in 6-well plates. Whole cell lysates from each triplicate sample were prepared by washing in cold buffered 1X PBS (GIBCO) and then lysed in RIPA buffer (Invitrogen) containing 1:100 protease inhibitors. After centrifugation (12,000 rpm, 15 min) at 4° C, protein supernatants were transferred into new tubes. Protein concentrations of the samples were determined with a bicinchoninic acid protein assay (Pierce). Lysates were normalized to 25 μg and then heated for 5 min (95° C) in Tris-Glycine SDS sample buffer and sample reducing agent (Invitrogen) prior to loading onto XCell4 SureLock™ Midi-Cell 10% SDS polyacrylamide gel electrophoresis system (Invitrogen) and blotted onto nitrocellulose using a Trans-Blot Cell Blotter (BioRad).

Membranes were then blocked with 5% non-fat milk in TBST (20 mM Tris, 150 mM NaCl, 0.05% Tween-20) for 30 min and incubated at 4° C overnight with primary antibodies p21 and α-Tubulin (both from Cell Signaling) at a 1:1000 dilution. Subsequently, the membranes were washed with TBST three times for 5 min and incubated with secondary antibodies (HRP-anti-rabbit or HRP-anti-mouse, both from Cell Signaling) at 1:2000 dilution for 1h at room temperature. Bound antibody was visualized by incubation with ECL solution (Pierce) for 1 min at room temperature and then imaged using the Azure 280 Western Blot Imaging System (Azure Biosystems). Protein bands were analyzed and quantified using the ImageJ software.

### Statistical analysis

Control and treated groups were compared using the analysis of variance (ANOVA) test. Results are presented as the mean value ± SEM of three independent experiments performed in triplicates. Statistical significance was determined by an unpaired two-tailed Student’s t-test in which a *P* < 0.05 was considered to indicate statistical significance. Data was processed using Microsoft Excel software.
